# A case of preeclampsia with severe features following septic shock and drug-induced acute kidney injury

**DOI:** 10.1515/crpm-2021-0093

**Published:** 2023-03-27

**Authors:** Emily M. Boyd, Michelle T. Nguyen, Brian Gordon, Richard H. Lee

**Affiliations:** Department of Anesthesia, University of California, San Francisco, CA, USA; Division of Maternal-Fetal Medicine, Department of Obstetrics and Gynecology, Keck School of Medicine of the University of Southern California, Los Angeles, CA, USA

**Keywords:** acute kidney injury, lithium, preeclampsia, psychosis, sepsis

## Abstract

**Objectives:**

This article outlines a process for differentiating preeclampsia from other potential causes of end-organ damage in a critically ill patient.

**Case Presentation:**

A patient in her early 30s, G2P1001 with intrauterine pregnancy at 12 weeks’ gestation was admitted seven months after delivering her first child due to new-onset psychosis and starvation ketosis. She was started on lithium for postpartum psychosis at 20 weeks’ gestation. She subsequently developed respiratory failure at 26 weeks’ gestation due to aspiration pneumonia in the setting of lithium toxicity, requiring admission to the intensive care unit (ICU) and intubation. She received antibiotics and vasopressors for suspected septic shock in addition to dialysis for lithium-induced acute kidney injury. One week after ICU admission, her sepsis resolved, her serum creatinine levels returned to normal, and her respiratory status improved. However, after vasopressors were discontinued, she suddenly developed persistently elevated blood pressures with proteinuria and elevated liver function tests (LFT’s). Due to concern for preeclampsia with severe features and rapidly increasing LFT’s, the patient underwent cesarean delivery at 27 weeks’ gestation.

**Conclusions:**

In a critically ill patient with multiple comorbidities, it can be difficult to diagnose preeclampsia using the standard criteria. It is important to exclude other potential etiologies, as a misdiagnosis can have potentially devastating consequences.

## Introduction

Preeclampsia (PE) is a hypertensive disorder that complicates 2–8% of pregnancies globally and leads to severe perinatal morbidity and mortality, including the morbidity and mortality associated with preterm birth [1]. The incidence of PE has increased by about 25% over the years of 1987 and 2004 [1]. Risk factors for developing PE include nulliparity, new paternity, excess placental volume (such as with hydatidiform moles and multifetal gestation), advanced maternal age, pre-existing chronic hypertension, pre-gestational and gestational diabetes, obesity, kidney disease, thrombophilia, systemic lupus erythematosus, antiphospholipid antibody syndrome or other vascular disorders, obstructive sleep apnea, and personal or family history of PE.

Clinical signs and symptoms of PE can include headache, vision changes, right upper quadrant pain, or shortness of breath, but these symptoms may be unreliable and nonspecific, and some women with PE may be asymptomatic. The American College of Obstetricians and Gynecologists (ACOG) provides a list of specific diagnostic criteria that includes both clinical symptoms and objective vital sign abnormalities and laboratory data [1]. As evidenced by these various criteria, PE can have multi-organ system consequences, including vascular, hematologic, hepatic, and renal effects.

Diagnosing PE using the standard criteria may not be straightforward because other obstetric and non-obstetric conditions can present with abnormal symptoms, vital signs, and laboratory values that mimic the presentation of PE. For example, patients with acute fatty liver of pregnancy often present with elevated liver function tests (LFT’s) and thrombocytopenia. As another example, patients with thrombotic thrombocytopenic purpura (TTP) or atypical hemolytic uremic syndrome (aHUS) often present with hemolytic anemia, thrombocytopenia, and elevated creatinine, with the creatinine elevation usually being more pronounced in aHUS than in TTP [2]. Patients with abnormal blood pressures and signs of end-organ damage in the setting of coexisting medical complications, such as sepsis or drug intoxication, pose a unique diagnostic challenge in which PE must be distinguished from other diagnoses as the main contributor to end-organ damage. We present the case of a patient with no known risk factors for hypertensive disease whose diagnosis of PE with severe features, based on severe-range blood pressures and elevated LFT’s, was confounded by several factors including pre-existing anemia of chronic disease, recent use of vasopressors for septic shock, recent acute kidney injury (AKI) due to lithium toxicity, and administration of potentially hepatotoxic medications.

## Case presentation

A patient in her early 30s, G2P1001 with intrauterine pregnancy at 12 weeks’ gestation was initially admitted to an inpatient psychiatric unit seven months after delivering her first child due to new-onset psychosis and starvation ketosis. According to her husband, she had been self-isolating, not eating, and not taking care of her infant in the weeks after the birth of her first child. Her past medical history included hypothyroidism, inflammatory bowel disease, and anemia of chronic disease, but no prior psychiatric illness. Her thyroid stimulating hormone (TSH) level confirmed appropriate levothyroxine dosing. Her psychotic symptoms did not improve after initial inpatient treatment with olanzapine, mirtazapine, and haloperidol, and she continued to deteriorate with ongoing refusal to eat and 10 kg weight loss, which was a 20% loss from her weight at presentation. Her pregnancy was also complicated by an ultrasound diagnosis of multiple fetal anomalies at 17- and 20-weeks’ gestation, including double outlet right ventricle, transposition of the great arteries, and intracranial ventriculomegaly. The patient and her husband (i.e., her surrogate decision-maker) were informed of these findings and counseled on management options including prenatal genetic screening tests and amniocentesis, which they declined. They were also counseled on the options of pregnancy continuation, adoption, and termination, and they decided to continue the pregnancy.

Postpartum psychosis was eventually determined to be the patient’s most likely psychiatric diagnosis, given that the onset of her mental illness occurred after the birth of her first child and then persisted into her current short-interval pregnancy. Therefore, at 20 weeks’ gestation, she was started on lithium, the first-line treatment for postpartum psychosis. At 26 weeks’ gestation, she developed symptoms of an upper respiratory infection and significantly decreased her oral intake of fluids due to pain with swallowing. Her dehydration led to volume depletion and impaired renal clearance of lithium, which precipitated lithium toxicity with serum lithium levels as high as 3.90 mmol/L (therapeutic range: 0.8–1.2 mmol/L) [3]. She then developed respiratory failure due to aspiration pneumonia in the setting of lithium toxicity, and she was admitted to the intensive care unit (ICU) where she was intubated, started dialysis for lithium-induced AKI, and received norepinephrine, midodrine, and piperacillin/tazobactam for presumed septic shock. Blood, respiratory, stool, and urine cultures ultimately came back negative for growth; however, all the cultures were collected after antibiotic therapy had already been initiated. One week after ICU admission her fevers resolved, her chest X-ray showed interval decrease in multifocal patchy airspace opacities, her white blood cell count down-trended from 81.8 K/cumm to 19.1 K/cumm, her lithium level decreased to <0.10 mmol/L, and her serum creatinine improved from 2.58 mg/dL to 0.64 mg/dL. However, at 27 weeks’ gestation, she developed multiple mild-range blood pressures more than 4 h apart and intermittent severe-range blood pressures, which persisted for two days after vasopressors were discontinued. On the same day when her blood pressures became elevated, she also developed elevated LFT’s (AST 58 U/L, ALT 36 U/L), which rapidly increased to AST 413 U/L, ALT 281 U/L over the next 24 h. Liver ultrasound showed hepatomegaly with an otherwise normal- appearing liver. Due to concern for medication side effect as the underlying etiology of her elevated LFT’s, piperacillin/tazobactam was discontinued, and her sedative was switched from Propofol to dexmedetomidine.

**Figure 1:**
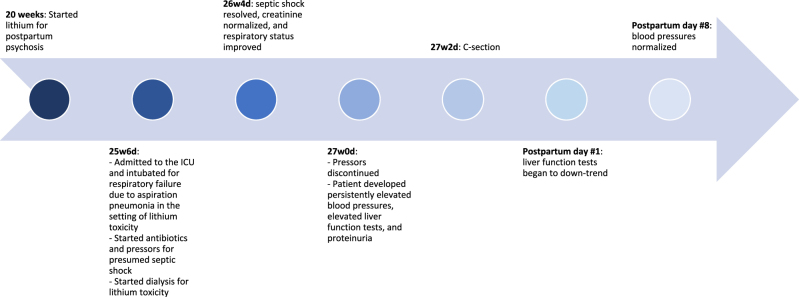
Timeline of major events during patient’s hospital course.

After these medication changes, her AST initially slightly down-trended to 323 U/L while ALT up-trended 346 U/L; however, her LFT’s subsequently increased again to AST 506 U/L, ALT 510 U/L. In addition to rapidly rising LFT’s, her labs were notable for an elevated LDH of 603 U/L, an elevated protein/creatinine ratio of 2.83, and up-trending platelet count in the 400s K/cumm. During this time, she continued to have intermittent mild-range blood pressures off vasopressors. However, she had normal serum creatinine, serum glucose level, and coagulation profile, and her peripheral blood smear did not show evidence of hemolysis. During extubation trial, she was noted to have rhythmic jerking of her bilateral upper extremities that was initially concerning for seizure activity vs. agitation; however, she subsequently had a normal electroencephalogram and computed tomography scan of the head. Due to the overall clinical picture concerning for PE with severe features, the patient received a betamethasone course for fetal lung maturity and intravenous magnesium sulfate for seizure prophylaxis, then underwent cesarean delivery at 27w2d. On postpartum day #1 her LFT’s began down-trending to AST 359 U/L, ALT 471 U/L, and by postpartum day #8 her blood pressures returned to normal with AST 19 U/L, ALT 96 U/L. At her outpatient postpartum checkup one month after delivery, she continued to have normal blood pressures with AST 15 U/L, ALT 15 U/L. Her laboratory values before and after delivery are summarized in Table 1, and a timeline of major events during her hospital course are summarized in Figure 1.

**Table 1:** Laboratory values before and after delivery.

Laboratory	26w5d	26w6d	27w0d	27w1d	27w2d	PPD#1	PPD#2	PPD#3	PPD#4	PPD#14	PPD#34
White blood cell count, K/cumm	24.1	22.3	27.1	20.2	24.8	23.1	20.0	18.7	13.0	11.3	10.3
Hemoglobin, g/dL	9.6	9.5	9.8	8.7	8.8	9.7	10.6	10.6	10.9	10.8	11.1
Platelet count, K/cumm	214	255	334	402	511	587	682	750	741	627	404
Aspartate transaminase, U/L	19	58	414	359	573	420	191	134	56	23	15
Alanine transaminase, U/L	16	36	237	319	503	481	365	332	241	36	15
Creatinine, mg/dL	0.73	0.90	0.89	0.64	0.66	0.67	0.56	0.63	0.55	0.61	0.57
Lactate dehydrogenase, U/L	N/A	N/A	713	659	988	721	428	344	299	N/A	N/A

PPD, postpartum day; N/A, not available ^a^Normal ranges, White blood cell count (4.5–10.0 K/cumm); Hemoglobin (11.6–15.4 g/dL); Platelet count (150–450 K/cumm); Aspartate transaminase (5–34 U/L); Alanine transaminase (0–55 U/L); Creatinine (0.6–1.1 mg/dl); Lactate dehydrogenase (125–220 U/L).

The neonate was small for gestational age with a birthweight of 840 g (3%), although the estimated fetal weight measured appropriate for gestational age on prenatal ultrasounds. The neonatal 1 min Apgar score was 0 and 5 min Apgar score was 1. The infant had respiratory distress syndrome and required extensive resuscitation including chest compressions for 10 min and intubation. Postnatal imaging confirmed multiple anomalies including septo-optic dysplasia, double inlet left ventricle with a highly underdeveloped right ventricle, and an interrupted aortic arch. The infant eventually deteriorated due to cardiac complications and demised after palliative extubation was performed on day of life #66.

## Discussion

Pregnant women with preeclampsia are at increased risk of iatrogenic preterm delivery, and preterm birth in turn is associated with neonatal consequences including respiratory distress, cerebral palsy, intraventricular hemorrhage, necrotizing enterocolitis, and neonatal death [4]. This case highlights the importance of identifying the correct underlying etiology of maternal hypertension and multi-organ damage, as cases due to non-obstetric causes can be treated without preterm delivery. The differential diagnosis for our patient’s elevated blood pressures and elevated LFT’s was broad and included vasopressors, medication side effects, ischemic hepatitis or “shock liver,” acute fatty liver of pregnancy, thrombotic thrombocytopenic purpura, and hemolytic uremic syndrome in addition to PE (Table 2). Almost all of these diagnoses could be reasonably ruled out for our patient; however, in many cases the clinical picture is not clear until after the decision to delivery is made, and the diagnosis of PE is clarified only once the patient improves after delivery. Ischemic hepatitis usually involves a rapid increase in transaminases (at least 20 times the upper limit of normal) and LDH [5]. The transaminase levels usually peak within 24 h, drop to about half within 24–72 h, and rapidly recover within seven to fourteen days [6]. This was not the case with our patient, whose LFT elevation was less drastic and did not start until about one week after the onset of suspected septic shock. Acute fatty liver was another potential etiology especially given her fever and leukocytosis, but the patient did not have hypoglycemia, hyperbilirubinemia, hyperammonemia, or an abnormal coagulation profile. Furthermore, her liver ultrasound showed only hepatomegaly without fatty infiltration, and her antithrombin III level was 95%, all of which decreased the likelihood of acute fatty liver [7]. TTP was lower on the differential as the patient had elevated platelets, not thrombocytopenia, and her ADAMTS13 level resulted at 75%. Similarly, aHUS was lower on the differential due to the patient’s elevated platelets and consistently normal creatinine after completing dialysis treatment for lithium toxicity.

**Table 2:** Differential diagnosis for patient’s elevated blood pressures and elevated liver function tests.

Differential diagnosis	Characteristic features
Preeclampsia with severe features	– RBC: hemolytic anemia
Hemolysis, elevated liver enzymes, low platelets (HELLP)	– Platelets: <100 K/cumm – LFT’s: >2x ULN – Creatinine: >1.1 mg/dL but <2.0 mg/dL – LDH: >600 U/L – Blood pressure: ↑ (peak at 48–72 h after delivery)
Thrombotic thrombocytopenic purpura	– RBC: hemolytic anemia – Platelets: <30 K/cumm – LFT’s: normal – Creatinine: <1.1 – ADAMTS13: <10%
Atypical hemolytic uremic syndrome	– RBC: <8.0 g/dL with hemolysis – Platelets: <150 K/cumm – Creatinine: >2.0 mg/dL – LDH ≥1000 U/L
Acute fatty liver of pregnancy	– Temperature: ≥100.4 °F – WBC: ↑ – RBC: normal – Platelets: <150 K/cumm – LFT’s: >2x/ULN – –Creatinine: >1.1 mg/dL – –Bilirubin: ↑ – –Ammonia: ↑ – PT/INR and PTT: ↑ – Glucose: ↓ – Antithrombin III: ↓ – Ultrasound: echogenic liver and/or ascites
Ischemic hepatitis/shock liver	– LFT’s: >20x ULN (peak in 24 h, drop by ½ in 24–72 h, and normalize in 7–14 days)
Piperacillin/tazobactam	– LFT’s: ↑ in 6–15% of patients
Propofol infusion syndrome	– LFT’s: ↑ (extremely rare) – Hepatomegaly – Metabolic acidosis – Rhabdomyolysis – Bradyarrhythmia
Vasopressors	– Blood pressure: ↑ until medication is tapered or discontinued

RBC, red blood cell; LFT’s, liver function tests; ULN, upper limit of normal; LDH, lactate dehydrogenase; ADAMTS13, a disintegrin and metalloproteinase with a thrombospondin type 1 motif, member 13; WBC, white blood cell; PT, prothrombin time; INR, international normalized ratio; PTT, partial thromboplastin time

Piperacillin/tazobactam may be associated with ALT elevations in 6–15% of patients, which resolve quickly upon stopping the antibiotic [8]. In addition, Propofol has been associated with a few cases of acute liver injury and “Propofol infusion syndrome,” which is characterized by cardiac bradyarrhythmia, metabolic acidosis, rhabdomyolysis, and an enlarged liver. However, Propofol-induced hepatotoxicity is extremely rare, and this sedative is generally not associated with marked elevations in LFT’s [9]. In our case, the patient was taken off both piperacillin/tazobactam and Propofol out of concern for drug-induced hepatic injury, which initially resulted in a slight decrease in AST, but both the AST and ALT rapidly increased again soon after. There was also concern for overly aggressive use of vasopressors; however, her blood pressures remained persistently elevated for two days even after discontinuing pressors.

Even after ruling out these differential diagnoses and potential medication side-effects, it was still difficult to make the diagnosis of PE because her clinical presentation was not fully consistent. Our patient did not have any known risk factors for PE. Although it is possible that the AKI predisposed the patient to PE, only chronic kidney disease or a history of AKI before pregnancy, as opposed to AKI during pregnancy, has been associated with higher risks of PE [10]. PE is a risk factor for sepsis; however, sepsis is not conversely a risk factor for PE [11]. Furthermore, PE is usually characterized by a decrease in platelet count, but her platelets up-trended to the 400 s K/cumm. Even at baseline, many of her lab values were already abnormal. For example, she had a low hemoglobin of 9.4 g/dL upon admission at 12 weeks’ gestation due to anemia of chronic disease, and her creatinine had recently been elevated due to lithium toxicity. Her proteinuria was difficult to interpret in the setting of recent lithium-induced AKI. Lastly, misdiagnosis of the patient’s LFT elevation as a severe feature of PE would be potentially devastating to the neonate at this point, as it would result in unnecessarily early delivery at 27 weeks’ gestation of an infant with multiple congenital anomalies whose prognosis would be significantly more impacted by prematurity.

Currently in the United States, the primary modality for preeclampsia screening is obtaining the patient’s history to elicit maternal and pregnancy characteristics and medical comorbidities that are classified by ACOG as “high” or “moderate” risk factors, which would warrant initiation of aspirin prophylaxis. However, this assessment of clinical factors has a sensitivity of only 41% in predicting preterm preeclampsia and an even lower sensitivity in predicting preeclampsia at any gestational age [12]. As previously mentioned, our patient screened negative for preeclampsia based solely on her clinical characteristics. Several biomarker screens have recently been investigated to potentially improve prediction of preeclampsia, including placental growth factor (PlGF) <100 pg/mL, soluble fms-like tyrosine kinase 1 (sFlt1):PlGF ratio >38, alpha fetoprotein (AFP):pregnancy-associated plasma protein A (PAPP-A) ratio >10, and a novel first trimester screen that combines mean arterial blood pressure, mean uterine artery resistance on doppler testing, and PlGF [12]. The first trimester combined screen appears to improve the sensitivity for predicting preterm preeclampsia to 82% but has several limitations, including 42.5% sensitivity in predicting term preeclampsia and the increased costs of blood testing and doppler testing [12]. First-trimester biomarker screening in our patient potentially could have enhanced prediction of preterm preeclampsia.

Ultimately, PE with severe features was determined to be the most likely diagnosis. There was a 1-week delay between onset of presumed septic shock and onset of LFT elevation, reducing the likelihood of any association between the two. The patient’s blood pressures did not improve after discontinuing vasopressors, and her LFT elevation did not improve after discontinuing Propofol and piperacillin/tazobactam. Although there were certain lab values that did not necessarily support a diagnosis of PE with severe features (i.e., elevated platelets and persistently normal creatinine after dialysis), the labs were overall not consistent with the alternative diagnoses either. The patient’s blood pressures and LFT’s both improved significantly following delivery, providing the most compelling support for our diagnosis of PE.

## Conclusions

Preeclampsia can often be diagnosed using the standard diagnostic criteria, but some patients with PE may not completely fit the typical criteria, or there may be multiple other explanations for the patients’ clinical and lab abnormalities. This case highlights how difficult the diagnosis of PE is, especially in critically ill patients with a complex medical picture whose signs of end-organ damage can potentially be attributed to other confounding problems or medication side-effects. The differential diagnoses for PE often have overlapping clinical findings and lab abnormalities. In these cases, it is important to look individually at each confounding factor and rule it in or out as a potential explanation for the patient’s presentation. This is especially important because a prompt diagnosis of worsening PE with severe features is vital as it can warrant preterm delivery to minimize the risks of maternal morbidity and mortality. However, a misdiagnosis of PE can lead to unnecessary preterm delivery with potentially devastating neonatal consequences.
